# Comparative effects of different exercise modalities on cognitive domains in older adults: a Bayesian network meta-analysis and meta-regression

**DOI:** 10.3389/fnut.2026.1797113

**Published:** 2026-05-29

**Authors:** Linlin Yang, Zhichao Yuan, Xiaoan Chen

**Affiliations:** 1 College of Sports Science, Jishou University, Jishou, Hunan, China; 2 China National Academy of Educational Sciences, Beijing, China

**Keywords:** Bayesian network meta-analysis, executive function, exercise intervention, memory, older adults

## Abstract

**Objective:**

To compare the effects of different exercise modalities on multiple cognitive domains in cognitively healthy older adults and examine associations between exercise dose parameters and cognitive benefits.

**Methods:**

Randomized controlled trials (RCTs) of exercise interventions in cognitively healthy older adults were systematically searched. Outcomes were classified into five domains: executive function, memory, verbal fluency, processing speed, and working memory. Random-effects pairwise meta-analyses were performed using standardized mean differences (SMDs). Prespecified subgroup analyses were conducted by participant and intervention characteristics. Linear random-effects meta-regressions examined frequency, session duration, intervention length, weekly dose, and total dose as continuous moderators. Bayesian network meta-analysis (NMA) was applied to compare and rank exercise modalities, distinguishing active and PCs.

**Results:**

Pairwise meta-analyses showed significant improvements in executive function (SMD = 0.31, 95% CI 0.18–0.44), memory (SMD = 0.26, 95% CI 0.14–0.38), and processing speed (SMD = 0.25, 95% CI 0.12–0.38), a borderline effect for working memory (SMD = 0.20, 95% CI 0.00–0.39), and no significant effect for verbal fluency. Meta-regression showed positive associations of training frequency and weekly dose with executive function, and of training frequency with processing speed. Bayesian NMA suggested aerobic exercise (AE) ranked higher for executive function and memory, while mind–body exercise (MBE) showed potential advantages for verbal fluency and working memory; however, most between-modality comparisons remained inconclusive.

**Conclusion:**

Exercise benefits multiple cognitive domains in cognitively healthy older adults, with more consistent effects on executive function and memory. AE appears promising, but further high-quality head-to-head trials with long-term follow-up are warranted.

**Systematic review registration:**

https://www.crd.york.ac.uk/PROSPERO/view/CRD420261290474, CRD420261290474.

## 1 Introduction

Against the backdrop of global population aging, maintaining cognitive health and reducing the risk of dementia have become central components of strategies for healthy aging (1). As the older population continues to expand, cognitive decline–related functional impairments and the growing demand for care pose substantial challenges to the sustainability of health and social care systems (2, 3). Accumulating evidence suggests that the transition from normal aging to mild cognitive impairment (MCI) and ultimately dementia is not an abrupt event, but rather a progressive process unfolding over many years or even decades. This trajectory may involve the accumulation of neuropathology, reduced efficiency of brain networks, and subtle yet measurable fluctuations in cognitive performance (4). Within biomarker-informed disease staging frameworks, Alzheimer’s disease–related pathological changes may begin long before the onset of clinical symptoms, making the pre-symptomatic stage a critical window for primary prevention and risk reduction interventions (5, 6). Given the lack of disease-modifying therapies that can fundamentally halt or reverse the underlying pathology of dementia, international guidelines and consensus statements emphasize the prioritization of early intervention targeting modifiable lifestyle factors to slow cognitive decline and reduce the future risk of MCI or dementia (7). Accordingly, individuals in the preclinical and prodromal phases have become key target populations for contemporary intervention studies and clinical trials (8, 9).

Among modifiable lifestyle factors, physical activity and structured exercise interventions have been widely regarded as key public health strategies to promote cognitive health and reduce dementia risk, given their favorable safety profile, accessibility, and scalability (7, 10–13). Exercise may confer cognitive benefits in later life through multiple biological pathways, including improving cardiometabolic and cerebrovascular function, enhancing neuroplasticity-related mechanisms, and modulating chronic inflammatory burden (14–18). However, the existing evidence remains heterogeneous and sometimes inconsistent, with effect sizes and domain-specific findings varying substantially across studies. Such discrepancies may be partly attributable to the considerable variability in exercise prescription parameters (e.g., frequency, intensity, session duration, and intervention length) as well as intervention modalities (16, 19). Importantly, cognition is not a unitary construct but comprises multiple relatively distinct domains, such as executive function, memory, verbal fluency, processing speed, and working memory, which differ in their sensitivity to aging and capacity for plasticity (20). Therefore, variations across exercise modalities in physiological stimulus characteristics and neuroregulatory demands suggest that their effects on specific cognitive domains may be modality-specific (18).

Although the evidence has been repeatedly examined in multiple systematic reviews and meta-analyses, it remains difficult to translate current findings into clear and actionable exercise prescription recommendations. First, most previous studies have relied primarily on pairwise meta-analyses, typically focusing on the overall effect of “exercise versus control,” thereby limiting direct comparisons of the relative effects across different exercise modalities (21, 22). Second, control conditions vary substantially across trials, and passive and AC are often pooled together, which may bias effect size estimates and reduce the clinical interpretability of the conclusions (21, 23). In addition, key dose parameters of exercise prescriptions (e.g., frequency, session duration, and intervention length) show considerable between-study variability, and prior evidence has largely remained at the level of subgroup comparisons, leaving the dose–response relationship insufficiently quantified (24). Meanwhile, existing studies have tended to emphasize global or composite cognitive outcomes, whereas domain-specific evidence for executive function and memory remains relatively limited, further constraining the development of targeted and precise exercise recommendations for distinct cognitive goals (25, 26). In this study, executive function was operationally conceptualized as higher-order cognitive control processes supporting goal-directed behavior, particularly cognitive flexibility, set-shifting, inhibitory control, problem solving, and task switching, as reflected by commonly used neuropsychological tasks such as the Trail Making Test Part B and the Wisconsin Card Sorting Test. Memory was operationally defined as performance related primarily to episodic memory, including learning, encoding, immediate recall, delayed recall, and recognition of verbal or visuospatial information, rather than procedural or semantic memory. Working memory was considered separately because it primarily involves the temporary maintenance and manipulation of information.

To address the above evidence gaps, this study aimed to systematically compare the effects of different exercise modalities on key cognitive domains in cognitively healthy older adults. Cognitive outcomes were categorized into five domains: executive function, memory, verbal fluency, processing speed, and working memory. In addition to conducting pairwise meta-analyses to estimate overall effects, we performed prespecified subgroup analyses to explore potential sources of heterogeneity and further applied meta-regression models to examine associations between key exercise dose parameters and effect sizes. To enable relative comparisons and ranking across multiple exercise modalities, we conducted a Bayesian NMA, in which control conditions were classified as active control (AC) and passive control (PC) to improve comparability and enhance the clinical interpretability of the findings. Collectively, these analyses are expected to provide evidence to inform domain-specific optimization of exercise prescriptions for cognitively healthy older adults.

## 2 Methods

### 2.1 Study design and registration

This meta-analysis was conducted in accordance with the PRISMA-NMA guidelines and was prospectively registered in PROSPERO (CRD: 420261290474).

### 2.2 Search strategy and study selection

A comprehensive literature search was performed in Web of Science (WOS), Scopus, PubMed, PsycINFO, the Cochrane Library, Embase, and SPORTDiscus from database inception to October 2025. To minimize the risk of missing eligible studies, the reference lists of relevant original articles and previous systematic reviews were also manually screened.

To ensure both sensitivity and specificity, a structured search strategy was developed. In PubMed, the search was conducted using a combination of Medical Subject Headings (MeSH) terms and free-text keywords. Study screening was conducted independently by two reviewers (YLL and YZC) based on titles/abstracts and full texts. Any disagreements were resolved through discussion, or adjudicated by a third author (CXA) when necessary. The complete search strategy is provided in Additional file 1.

### 2.3 Eligibility criteria

We included only RCTs investigating exercise interventions in cognitively healthy older adults. Trials with incomplete outcome reporting or insufficient data for effect size estimation were excluded. Detailed inclusion and exclusion criteria are presented in Table 1.

**Table 1:** Eligibility criteria.

Domain (PICOS)	Inclusion criteria	Exclusion criteria
P (Participants)	Older adults aged ≥60 years; cognitively healthy at baseline (i.e., no diagnosis of MCI, dementia, or Alzheimer’s disease); recruited from community settings, residential care facilities, or health examination cohorts.	(1) Participants with a confirmed diagnosis of MCI or dementia/Alzheimer’s disease; (2) populations with cognitive impairment primarily due to neurological or psychiatric disorders (e.g., Parkinson’s disease, post-stroke cognitive impairment); (3) non-older populations (<60 years).
I (Interventions)	The experimental group received structured exercise intervention as the sole intervention. Eligible modalities included, but were not limited to: AE, resistance training (RT), MBE, multicomponent exercise (MCE), and balance training (BT). Intervention duration was ≥4 weeks. Programs could be delivered in group-based or home-based settings, and may be supervised or unsupervised.	(1) Exercise combined with other interventions (e.g., exercise plus nutrition/diet/supplements, cognitive training, or pharmacotherapy); (2) insufficient description of exercise protocols or inability to classify exercise modality; (3) non-structured lifestyle advice (e.g., general recommendations to “exercise more” without a prespecified program).
C (Comparators)	Comparators included: (1) no intervention/usual lifestyle/usual care; (2) health education, social activities, stretching/relaxation; (3) other structured exercise modalities (allowing direct head-to-head comparisons and serving as nodes for NMA).	Control groups that included additional interventions such as medication or cognitive training, or comparator conditions that were insufficiently described and could not be classified.
O (Outcomes)	Trials reported at least one cognitive outcome domain eligible for meta-analysis, including executive function, memory, verbal fluency, processing speed, and working memory, with sufficient data to calculate effect sizes.	(1) Lack of extractable quantitative data for synthesis; (2) outcomes reported only as subjective measures or in formats that could not be converted for meta-analysis.
S (Study design)	RCTs published as full-text articles in peer-reviewed English-language journals.	(1) Non-original publications: reviews/systematic reviews/meta-analyses, editorials, letters, case reports, conference abstracts, or unpublished manuscripts; (2) non-English publications; (3) non-randomized designs (e.g., cohort, cross-sectional, quasi-experimental, or pre–post single-arm studies).

### 2.4 Data extraction

Two reviewers (YLL and YZC) independently extracted data using a standardized form. Any discrepancies were resolved through discussion with a third author (CXA). The following information was collected: first author, publication year, country, sample size, age, sex, participant characteristics, body mass index, years of education, exercise modality, session duration, exercise intensity, exercise frequency, intervention length, and cognitive outcomes (executive function, memory, verbal fluency, processing speed, and working memory). For quantitative synthesis, post-intervention means and standard deviations were preferentially extracted. When required data were missing or incomplete, we attempted to contact the corresponding authors for clarification. If the information could not be obtained, missing values were estimated based on available data.

### 2.5 Risk of bias and quality assessment

The methodological quality of the included studies was independently assessed by two reviewers (YLL and CXA). Risk of bias for RCTs was evaluated using the Cochrane Risk of Bias 2 (RoB 2) tool, which covers five domains: (1) bias arising from the randomization process, (2) bias due to deviations from intended interventions, (3) bias due to missing outcome data, (4) bias in measurement of the outcome, and (5) bias in selection of the reported result. In addition, the PEDro scale (11 items, total score range 0–10) was applied to further evaluate study design quality and methodological rigor, including key aspects such as blinding, allocation concealment, and baseline comparability. Any discrepancies were resolved by discussion; if consensus could not be reached, a third reviewer (YZC) adjudicated. Finally, confidence in the network estimates was assessed using the CINeMA online platform (27, 28), and the assessment reports and visual outputs were exported for presentation.

### 2.6 Statistical analysis

Given that some dose groups had relatively small sample sizes and evidence for certain exercise modality comparisons was limited, we performed the analyses using a Bayesian framework, which allows flexible modeling of complex evidence networks and provides robust inference under sparse data conditions. SMDs were calculated using Hedges’ g to harmonize continuous outcomes measured on different scales. Effect estimates are reported with 95% credible intervals (CrIs). All statistical analyses were conducted in R software (version 4.5.1; Additional file 2).

#### 2.6.1 Data processing

All raw data were first used to calculate SMDs and their standard errors (SEs) in STATA 14. The effect sizes were then formatted as relative effects (diff) and corresponding standard errors (std.err) and imported into R for subsequent analyses. Although the gemtc package uses the mean difference (MD) as the default effect measure for continuous outcomes, the underlying contrast-based model is not sensitive to the measurement scale. Therefore, the pre-calculated SMDs were entered as the effect size input to perform Bayesian NMA based on SMDs. For multi-arm trials, to ensure appropriate variance specification and model convergence, we applied standard approaches to estimate the SEs of the reference arm. Because covariance information is rarely reported, we assumed a correlation coefficient of *ρ* = 0.5 between effect estimates, and calculated the baseline standard error using recommended formulas. When the same intervention modality was reported in multiple arms within a single study, data were combined using standard methods to obtain a single pooled estimate (mean ± SD) for meta-analysis.

#### 2.6.2 Pairwise meta-analysis

All statistical analyses were conducted in R (version 4.5.1). A two-sided *p* < 0.05 was considered statistically significant. SMDs were used as the effect size metric, and pooled estimates were reported with 95% confidence intervals (CIs). Between-study heterogeneity was quantified using the I^2^ statistic, with I^2^ > 50% indicating substantial heterogeneity. Forest plots were generated to present individual study estimates and the pooled effect. Prespecified subgroup analyses were performed according to exercise modality, intervention length, training frequency, session duration, cognitive outcome domain, and intervention setting. To assess the robustness of the findings, sensitivity analyses were conducted using a leave-one-out approach. Publication bias was evaluated using Egger’s regression test and contour-enhanced funnel plots. If funnel plot asymmetry was detected, the pooled effect size was further adjusted using the Duval and Tweedie trim-and-fill method, and the adjusted funnel plot was generated accordingly.

#### 2.6.3 Subgroup analyses

To explore potential effect modifiers and explain between-study heterogeneity, prespecified subgroup analyses were conducted based on the primary analyses. Effect sizes were stratified by geographic region (Europe, Americas, Asia), age (<70 vs. ≥ 70 years), years of education (<12 vs. ≥ 12 years), intervention setting (highly supervised, largely unsupervised, or mixed), key exercise dose parameters (frequency: ≤2 sessions/week, 3–4 sessions/week, ≥5 sessions/week; session duration: <45 min, 45–60 min, >60 min; intervention length: ≤12 weeks, 13–24 weeks, >24 weeks), exercise modality (aerobic, resistance, multicomponent, mind–body, or balance training), and type of control condition (PC vs. AC). These subgroup comparisons were performed to evaluate whether the effects of exercise on cognitive outcomes differed across participant characteristics and intervention features.

#### 2.6.4 Bayesian network meta-analysis

A Bayesian NMA was conducted using a random-effects consistency model to compare the relative effects of different exercise modalities on cognitive outcomes. All analyses were performed in R (version 4.5.1) using the gemtc package interfaced with JAGS for MCMC sampling. Network geometry and connectivity were first examined using network plots. Global consistency was assessed by comparing the consistency model with an inconsistency model based on the unrelated mean effects (UME) approach using the deviance information criterion (DIC). Local inconsistency was further evaluated using the node-splitting method. Four independent chains were run in parallel, with 20,000 adaptation iterations (burn-in) followed by 50,000 sampling iterations (thin = 1). Weakly informative priors (default settings in gemtc) were applied for treatment effects and between-study heterogeneity parameters. Model convergence was assessed using the Gelman–Rubin diagnostic, with the potential scale reduction factor (PSRF) approaching 1.00, complemented by visual inspection of trace plots. Model fit was evaluated using DIC and residual deviance checks. Treatment effects were expressed as SMDs with 95% CrIs. In addition, ranking probabilities were derived from posterior distributions, and SUCRA values were calculated to summarize treatment ranking.

## 3 Results

### 3.1 Study selection

**Figure 1:**
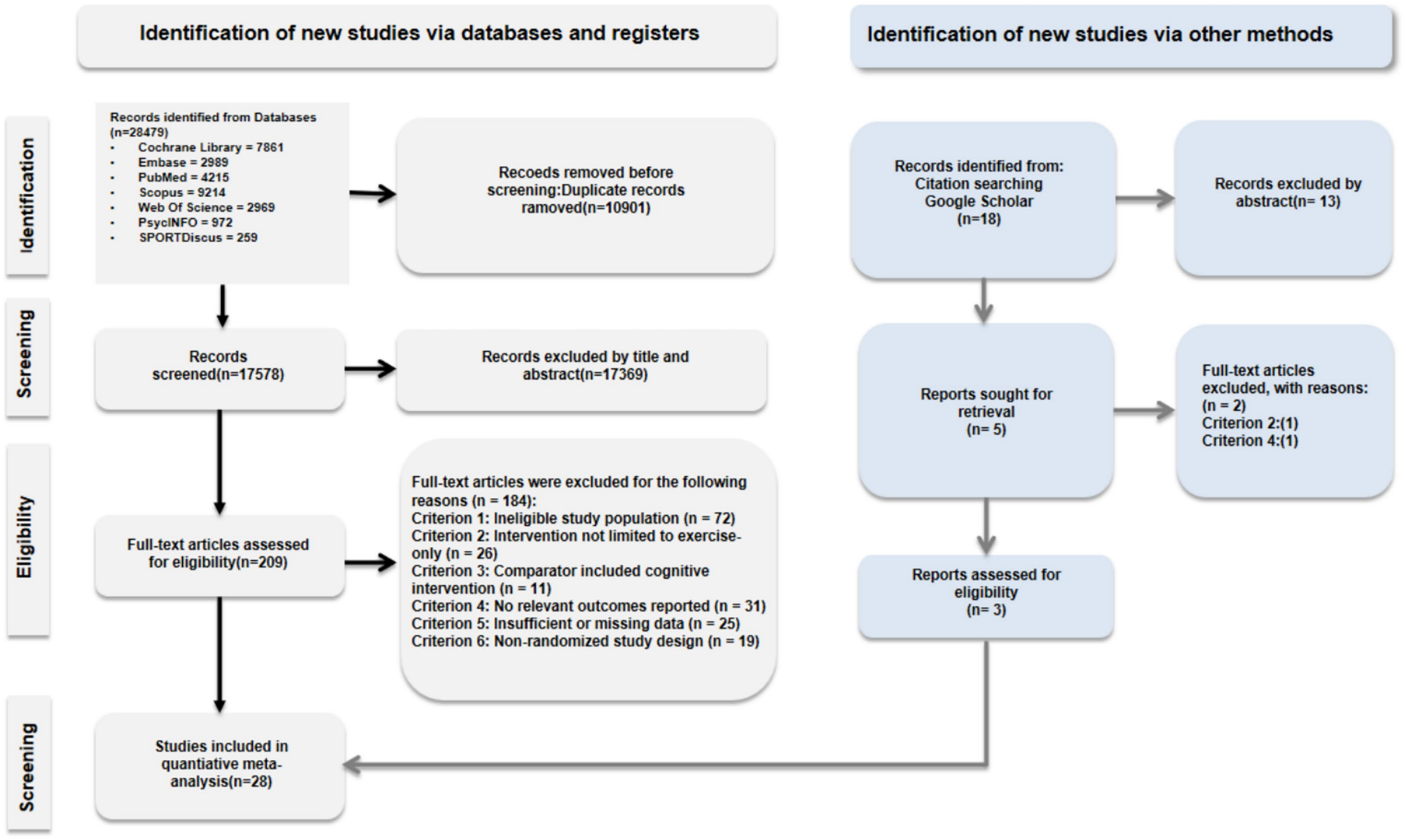
Flow diagram of study selection.

A total of 28,479 records were identified across seven databases (WOS, Scopus, PubMed, PsycINFO, the Cochrane Library, Embase, and SPORTDiscus). After removing 10,901 duplicates, 17,369 records were excluded during title and abstract screening according to the predefined inclusion and exclusion criteria. Full-text assessment led to the exclusion of 184 studies. In addition, 3 eligible study were identified through Google Scholar and citation tracking. Ultimately, 28 studies were included in the final analysis (Figure 1).

### 3.2 Study characteristics

TA total of 28 trials were included. Comprising 1,682 participants, with 1,008 assigned to the exercise intervention groups and 674 to the control groups. The trials were conducted across 15 countries or regions. All participants were cognitively healthy older adults aged ≥60 years, with reported age ranges generally spanning approximately 62–84 years.

Five exercise modalities were investigated: AE (29–46), RT (37, 47), MCE (39, 48–51), MBE (45, 52–55), and BT (37). Notably, four studies included multiple intervention arms (37, 45, 46, 54), and one study provided direct head-to-head comparisons between different interventions (39).

Interventions were typically delivered 1–7 sessions per week, with a session duration of 15–90 min, and an intervention length ranging from 8 to 60 weeks. Control conditions included usual lifestyle/no exercise, no intervention, health education programs, and low-intensity stretching or relaxation activities. A wide range of cognitive assessment instruments was used across studies, covering the domains of executive function, memory, verbal fluency, processing speed, and working memory; detailed outcome measures are provided in Additional files 3, 4.

### 3.3 Risk of bias and study quality

According to the RoB 2 assessment, one study was judged to be at low risk of bias overall (50), 12 studies raised some concerns (29–31, 34, 38, 40, 42, 43, 45, 49, 52, 55), and 15 studies were rated as having high risk of bias (32, 33, 35–37, 39, 41, 44, 46–48, 51, 53–55) (Additional file 5).

Based on the PEDro scale, 10 studies were classified as high quality (score ≥6) (29–31, 33, 38, 41, 43, 45, 49, 50), and 18 studies were of moderate quality (score 4–5) (32, 34–37, 39, 40, 42, 44, 46–48, 51–55), with no studies rated as low quality. Overall, the included trials showed moderate methodological quality, with generally acceptable randomization, baseline comparability, and outcome completeness, although blinding was frequently not feasible in exercise intervention trials (Additional file 6).

Confidence in the network estimates was evaluated using the CINeMA framework (Additional file 7). Overall confidence ratings ranged from high to very low, with downgrading mainly driven by imprecision and within-study bias. Heterogeneity affected several comparisons, particularly for executive function and processing speed, while incoherence was most evident for working memory.

### 3.4 Pairwise meta-analysis

#### 3.4.1 Main findings

**Figure 2:**
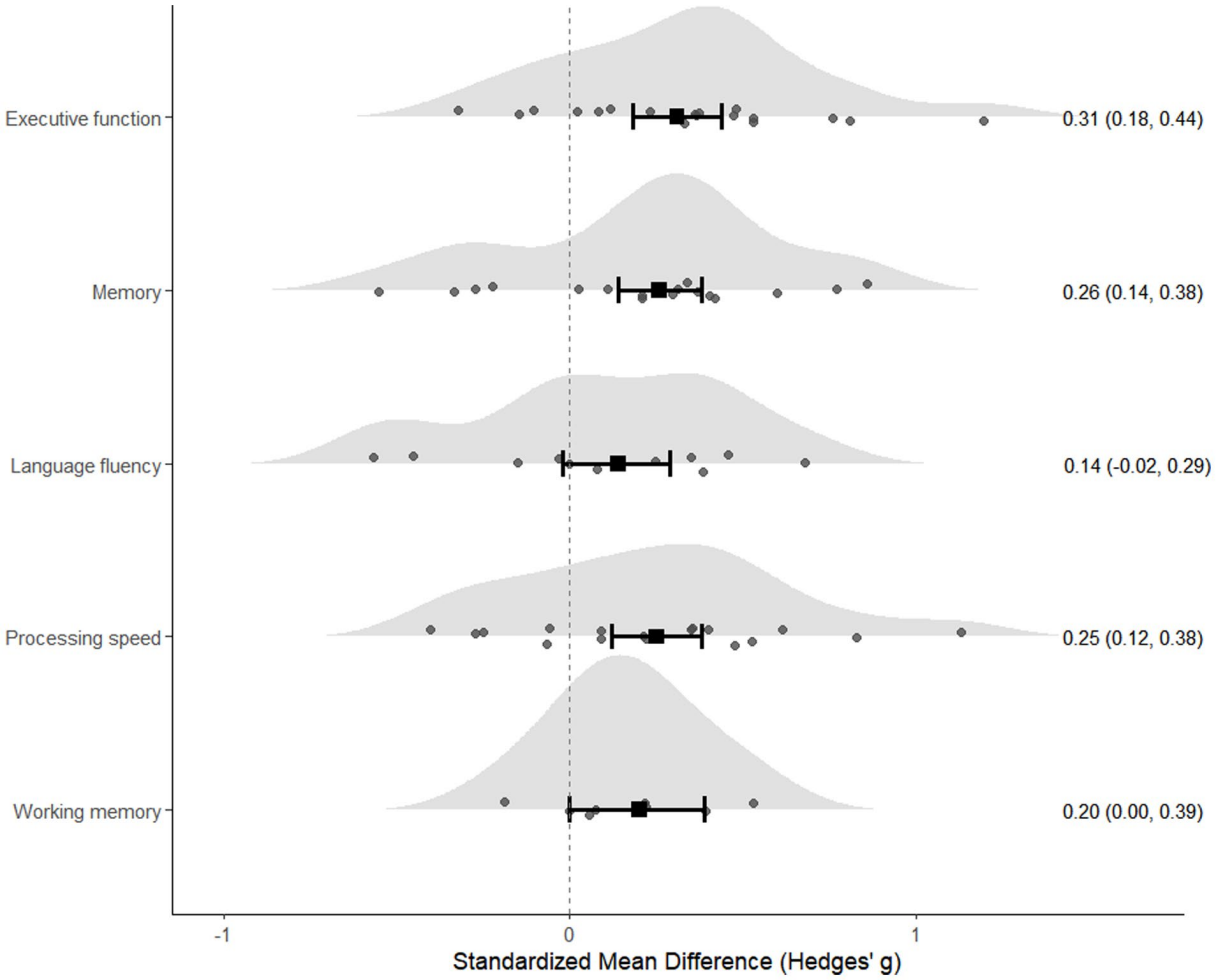
Forest plot of the pairwise meta-analysis.

In the pairwise meta-analyses, exercise interventions showed statistically significant improvements in most cognitive domains compared with controls. Exercise significantly improved executive function (SMD = 0.31, 95% CI 0.18–0.44, Z = 4.79, *p* < 0.0001; I^2^ = 29.3%) and memory (SMD = 0.26, 95% CI 0.14–0.38, Z = 4.09, *p* < 0.0001; I^2^ = 21.3%), with low between-study heterogeneity for both outcomes. Exercise also enhanced processing speed (SMD = 0.25, 95% CI 0.12–0.38, Z = 3.84, *p* = 0.0001; I^2^ = 43.9%). For working memory, exercise demonstrated a borderline significant improvement (SMD = 0.20, 95% CI 0.00–0.39, Z = 1.99, *p* = 0.047), with no evidence of heterogeneity (I^2^ = 0%). In contrast, no significant effect was observed for verbal fluency (Figure 2; Additional file 8).

#### 3.4.2 Heterogeneity, sensitivity analyses, and publication bias

To evaluate the robustness of the findings and potential publication bias, leave-one-out sensitivity analyses were conducted for each outcome, complemented by funnel plots, Egger’s regression tests, and the trim-and-fill method. The results for executive function and memory remained stable after sequential exclusion of individual studies, and Egger’s tests were not significant (*p* = 0.604 and *p* = 0.373, respectively). Although trim-and-fill suggested a small number of potentially missing studies (two imputed for each outcome), the adjusted pooled effects did not materially change. For verbal fluency, the pooled effect was small and its statistical significance varied across leave-one-out analyses, indicating limited robustness (Egger *p* = 0.564; one study imputed), and the results should be interpreted with caution. The findings for processing speed were stable, with no evidence of publication bias (Egger *p* = 0.810) and no imputed studies suggested by trim-and-fill. For working memory, the direction of effect was generally consistent but the pooled effect was borderline significant, and statistical significance fluctuated in sensitivity analyses. Funnel plot asymmetry was observed, and trim-and-fill indicated potential missing studies (four imputed; Egger *p* = 0.284), suggesting that this outcome may be influenced by small-study effects (Additional files 10–12).

### 3.5 Subgroup analyses

Prespecified subgroup analyses were conducted for all five cognitive outcomes according to region, age, years of education, intervention setting, key exercise prescription parameters (frequency, session duration, and intervention length), exercise modality, and type of control condition. Overall, subgroup findings suggested that responses to exercise may differ across cognitive domains. Improvements in executive function were relatively more consistent across subgroups. More stable positive effects were observed in studies with supervised interventions, moderate-to-high training frequency (≥3 sessions/week), session durations of 45–60 min, and intervention length ≤24 weeks. In terms of modality, both AE and MBE showed clearer improvement signals.

For memory, beneficial effects were mainly observed in studies conducted in Asia, those implementing supervised interventions, those with a longer intervention duration (24 weeks), and trials involving multicomponent exercise, whereas benefits were not consistently evident across all participant strata. Because the included trials used different memory tests, memory was analyzed as a cognitive domain using standardized effect sizes rather than as a composite score derived from identical memory measures; these measures primarily reflected episodic memory-related performance, including learning, immediate recall, delayed recall, and recognition. In contrast, verbal fluency and processing speed did not demonstrate consistent statistically significant improvements across most subgroups. Only limited positive signals were observed in a few strata (e.g., Asian studies, session duration of 45–60 min, and shorter intervention periods), suggesting considerable uncertainty. Subgroup results for working memory were also unstable: apart from the supervised intervention subgroup showing a significant improvement, most other strata did not reach statistical significance. Although the MBE subgroup yielded a relatively larger effect size, this finding should be interpreted cautiously due to the small number of available studies and wide CIs. Given the limited number of studies within several subgroups and the resulting imprecision, these subgroup findings should be considered exploratory and mainly hypothesis-generating rather than definitive (Additional file 9).

### 3.6 Meta-regression analyses

To explore potential dose–response relationships between exercise dose and cognitive benefits, we performed linear random-effects meta-regression analyses for the five cognitive outcomes, using training frequency, session duration, intervention length, weekly dose, and total dose as continuous moderators. The results showed a significant linear association between training frequency and effect size for executive function (linear slope test *p* = 0.025). In addition, weekly dose was positively associated with executive function improvements (linear slope test *p* = 0.032), suggesting that greater weekly training time may be related to larger gains in executive function. For processing speed, a significant linear association between training frequency and effect size was also observed (linear slope test *p* = 0.004). No statistically significant linear associations were detected for the remaining cognitive outcomes with respect to weekly dose or total dose (all *p* > 0.05). Overall, evidence for dose–response patterns was mainly confined to executive function, and these meta-regression findings should be considered exploratory (Additional file 13).

### 3.7 Bayesian network meta-analysis

#### 3.7.1 Network geometry and connectivity

**Figure 3:**
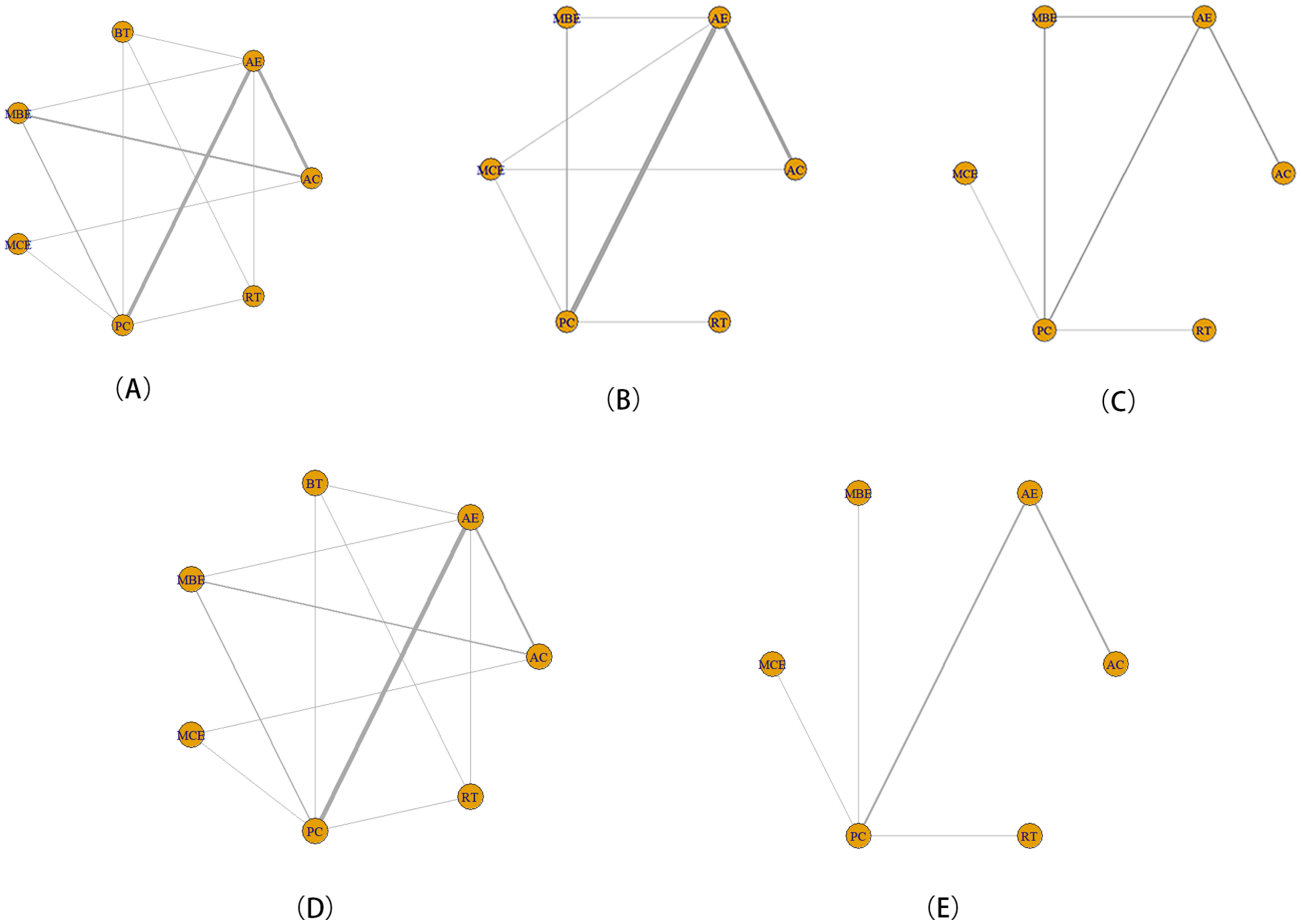
Network plots for each cognitive outcome. **(A)** Executive function; **(B)** Memory; **(C)** Verbal fluency; **(D)** Processing speed; **(E)** Working memory. AE, aerobic exercise; RT, resistance training; MCE, multicomponent exercise; MBE, mind–body exercise; BT, balance training; AC, active control; PC, passive control.

Control conditions were classified as AC and PC. Under the Bayesian random-effects consistency model, evidence networks were constructed based on direct comparisons from the included trials, and network plots were used to assess connectivity for each outcome (Figure 3). Overall, all networks included nodes for PC, AC, and multiple exercise modalities; however, network structures varied across outcomes. Some outcomes formed multiple closed loops with relatively well-connected networks, whereas others were comparatively sparse, with direct evidence largely concentrated on comparisons between exercise interventions and PC/AC. This pattern suggests limited indirect evidence and may increase uncertainty in effect estimation and treatment ranking. MCMC convergence was evaluated using trace plots and the Gelman–Rubin diagnostic, with all PSRF values approaching 1.00, indicating satisfactory convergence (Additional files 14, 18–22).

#### 3.7.2 Assessment of inconsistency

To evaluate network consistency, we fitted both a random-effects consistency model and an unrelated mean effects (UME) model, and compared the deviance information criterion (DIC), effective number of parameters (pD), and residual deviance (D). The difference in DIC between the consistency and UME models was <3, suggesting no evidence of statistically significant global inconsistency. Node-splitting analyses further indicated that, except for the comparison between MBE and AE in executive function (*p* = 0.033), no significant disagreement between direct and indirect evidence was detected for the remaining comparisons (all *p* > 0.05). Therefore, the random-effects consistency model was adopted as the primary model for the main analyses (Additional file 15).

#### 3.7.3 Main NMA findings

**Figure 4:**
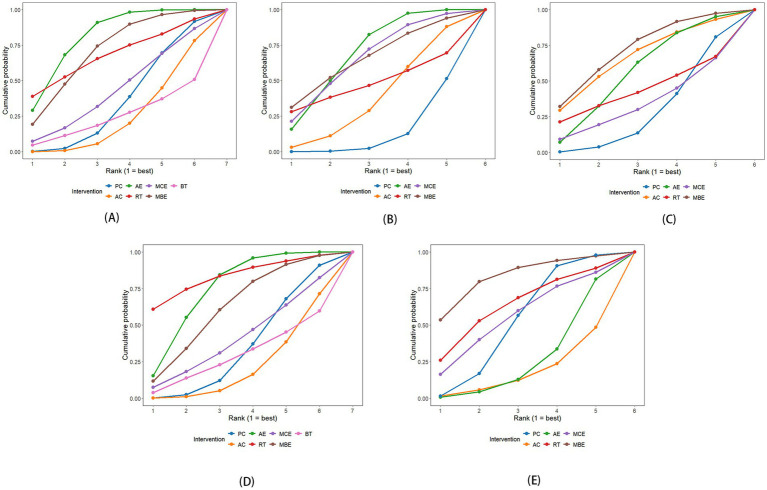
SUCRA plots for each cognitive outcome. **(A)** Executive function; **(B)** Memory; **(C)** Verbal fluency; **(D)** Processing speed; **(E)** Working memory.

For executive function, cumulative ranking probabilities suggested that AE ranked highest, followed by MBE. Although most pairwise comparisons in the league table had 95% CrIs crossing the null, AE showed a favorable trend compared with PC (SMD = 0.36, 95% CrI 0.00–0.77) and AC (SMD = 0.45, 95% CrI 0.07–0.88). For memory, AE also demonstrated the highest ranking probability, followed by MCE and MBE. While differences between most exercise modalities remained uncertain, AE showed a more consistent benefit compared with PC (SMD = 0.33, 95% CrI 0.06–0.62). For verbal fluency, processing speed, and working memory, ranking probabilities suggested that MBE (verbal fluency/working memory) and RT and AE (processing speed) may be among the top-ranked interventions. However, no clear statistically significant differences were observed in the league tables for these outcomes (all 95% CrIs crossed 0), indicating limited evidence to establish definitive superiority among exercise modalities. Therefore, these rankings should be interpreted as exploratory and with caution (Figure 4; Additional files 16, 17).

## 4 Discussion

By integrating evidence from pairwise meta-analyses, Bayesian NMA, and meta-regression, this study systematically evaluated the effects of exercise interventions on multiple cognitive domains in cognitively healthy older adults. Our findings not only help identify potentially optimal exercise strategies across cognitive domains, but also highlight intervention- and population-specific patterns, which may contribute to improving the precision of exercise prescription in aging societies.

### 4.1 Effects of exercise interventions on executive function

In cognitively healthy older adults, our pairwise meta-analysis demonstrated a consistent benefit of exercise on executive function (SMD ≈ 0.31), suggesting that executive function may be one of the cognitive domains most sensitive to exercise-related stimulation (56–58). Previous research has likewise indicated that executive function often responds more robustly to AE, potentially through exercise-induced enhancement of brain network integration and functional connectivity, thereby improving prefrontal cortex–related processes such as attentional control and cognitive flexibility (59).

To further interpret between-study variability, subgroup analyses provided additional insights. Improvements in executive function were more consistently observed in studies with more standardized implementation, greater supervision, and more clearly defined prescription components, suggesting that adherence and implementation quality may be key moderators of the reproducibility of executive function gains. Specifically, more stable positive effects were found in trials with higher training frequency (≥3 sessions/week), moderate session duration (45–60 min), and short- to mid-term intervention length (≤24 weeks), and clearer benefit signals were also noted in both AE and MBE subgroups. These findings are in line with previous network meta-analytic evidence, indicating that regular training frequency and an appropriate session duration may be favorable for improving executive function, while regional and population characteristics may also modify the magnitude of effects (60).

At the dose level, our meta-regression further suggested positive linear associations between executive function improvements and both training frequency and weekly dose, implying that regular stimulus exposure and weekly training load may play an important role in shaping gains in executive control (61). However, prior evidence regarding exercise dose–response relationships remains inconclusive, and the observed associations may be influenced by differences in trial design, comparator conditions, and incomplete reporting of prescription parameters. Therefore, these findings warrant further confirmation in higher-quality trials with more standardized and well-reported exercise prescriptions.

Meanwhile, the Bayesian NMA provided complementary evidence on potential differences across exercise modalities within a relative comparative framework. Ranking probabilities suggested that AE and MBE tended to rank among the top interventions, although substantial uncertainty remained across most between-modality comparisons. Thus, these rankings should be interpreted cautiously and primarily as exploratory signals. Consistent with this, previous reviews have reported relatively stable positive effects of AE on executive function (62, 63), and MBE may offer advantages in specific components of executive function (e.g., task switching and working memory–related processes) in some contexts (60).

### 4.2 Effects of exercise interventions on memory

Our pairwise meta-analysis showed that exercise interventions significantly improved memory in cognitively healthy older adults (SMD ≈ 0.26), with low between-study heterogeneity, indicating relatively consistent overall benefits in this domain. In this section, memory primarily refers to episodic memory-related performance, including learning, immediate recall, delayed recall, and recognition of verbal or visuospatial information, as assessed by the memory outcomes included in the reviewed trials. Subgroup analyses further suggested that memory improvements were more likely to be observed among participants who were older (≥70 years) and had fewer years of education (<12 years). More pronounced effects were also found in studies featuring highly supervised interventions, longer intervention durations (24 weeks), and longer session durations (60 min), implying that memory benefits may depend more strongly on sustained and accumulated stimulus exposure as well as stable implementation quality.

Although subgroup findings indicated that prescription structure may influence effect magnitude, our meta-regression did not identify statistically significant linear associations between dose parameters and effect sizes. From a relative comparative perspective, the Bayesian NMA suggested potential differences across exercise modalities for memory outcomes. Ranking probabilities indicated that AE and multicomponent exercise (MCE) tended to rank among the top interventions; however, only AE showed a more consistent benefit signal compared with controls in the league table, suggesting that current network evidence remains insufficient to establish a definitive hierarchy across modalities.

Importantly, the favorable trend for AE aligns with previous evidence. Prior network meta-analyses have also suggested that AE yields relatively stable positive effects on memory outcomes. Nagamatsu reported a significant association between post-intervention spatial memory performance and overall physical function in the aerobic training group (64). Han further indicated that AE was among the most effective approaches for enhancing memory (SMD = 0.42) (60). Moreover, Erickson et al. demonstrated that AE training increased anterior hippocampal volume in older adults and was accompanied by improvements in spatial memory, with hippocampal volume increases being correlated with elevated serum levels of BDNF, highlighting the potential involvement of neuroplasticity pathways in exercise-related memory benefits (15). Overall, current evidence supports a clear beneficial effect of exercise interventions on memory, particularly episodic memory-related performance involving learning, recall, recognition, and spatial memory, and Bayesian network findings further suggest that AE may provide relatively more stable benefits. Nevertheless, the optimal prescription profiles across different modalities require further validation through rigorously designed randomized trials with sufficient direct head-to-head comparisons.

### 4.3 Effects of exercise interventions on verbal fluency, processing speed, and working memory

Overall, the magnitude of exercise-related benefits observed for verbal fluency, processing speed, and working memory was relatively small, and the stability of findings was limited across these outcomes. For verbal fluency, the pairwise meta-analysis did not reveal a significant improvement, consistent with previous evidence in similar populations (65). However, subgroup analyses suggested that trials with session durations of 45–60 min and intervention length ≤12 weeks may exhibit potential benefit signals, indicating that verbal fluency may be more responsive to short-term and relatively concentrated training stimuli.

For processing speed, exercise interventions produced a significant improvement (SMD ≈ 0.25), although heterogeneity was substantial. Prior evidence indicates that TMT-A may be one of the cognitive measures that responds more consistently to exercise interventions (66). In line with this, our meta-regression identified a significant positive linear association between training frequency and effect size, suggesting that regular training exposure may represent a key prescription component for processing speed benefits.

For working memory, only a borderline significant improvement with a small effect size was observed (SMD ≈ 0.20), and the overall strength of evidence was limited. This is consistent with prior reviews reporting modest and unstable effects for working memory outcomes (67). Benefits were mainly observed in highly supervised interventions, further suggesting that adherence and implementation quality may influence the reproducibility of effects in this domain (61).

Ranking probabilities from the Bayesian NMA suggested that MBE may rank relatively higher for verbal fluency (45, 68) and working memory (69), whereas AE and RT may rank among the leading interventions for processing speed (70). However, league table comparisons did not demonstrate clear statistically significant differences, indicating that current evidence remains insufficient to support a robust modality hierarchy, and further high-quality randomized trials with direct comparisons are warranted.

### 4.4 Mechanistic considerations

The cognitive benefits of AE in older adults are unlikely to be driven by a single mechanism; rather, they may arise from multiple interrelated physiological and psychological pathways. First, AE can improve cardiorespiratory fitness and enhance cerebral perfusion, endothelial function, and regional cerebral blood flow (71, 72). It may also reduce insulin resistance and cardiometabolic risk burden, thereby providing a more favorable metabolic and vascular milieu for prefrontal cortex–related executive control and information-processing functions (73). At the molecular and cellular levels, AE may stimulate the release of neurotrophic factors such as BDNF, NGF, and IGF-1, promoting synaptic plasticity and long-term potentiation (LTP) (74, 75). In addition, modulation of neurotransmitter systems (e.g., dopamine) may facilitate adaptive plastic changes in hippocampal and prefrontal networks (76). Structurally, long-term AE has been associated with increased hippocampal and medial temporal lobe volumes (15) and improvements in cortical thickness, providing potential neurobiological support for enhanced memory and learning. Furthermore, regular physical activity may alleviate stress, depressive and anxiety symptoms, improve affective well-being, and enhance cognitive resilience, which may further consolidate cognitive gains through psychological pathways (77, 78). Overall, AE may promote improvements in executive function and memory through integrated effects on cardiovascular–metabolic optimization, neuroplasticity enhancement, and emotional regulation. The magnitude of benefits may be influenced by training intensity and regularity and may exhibit a certain degree of domain specificity.

### 4.5 Limitations

Several limitations of this study should be acknowledged. First, the number of included trials was limited for certain exercise modalities and within several subgroup strata, resulting in reduced precision. Given multiple subgroup comparisons, these findings should be considered hypothesis-generating rather than definitive, particularly for outcomes with relatively weaker evidence such as verbal fluency and working memory. Second, the intervention durations of the included studies were generally short, and long-term follow-up assessments were scarce, preventing conclusions regarding the sustainability of exercise-related cognitive benefits over time. Third, evidence from meta-regression analyses regarding dose–response relationships was mainly observed for executive function and processing speed, whereas no stable linear associations were detected for memory, verbal fluency, or working memory. Fourth, although ranking probabilities from the Bayesian NMA suggested that AE, RT, or MBE may rank among the leading interventions for certain outcomes, most league table comparisons had 95% CrIs crossing the null, indicating that the current network evidence remains insufficient to establish a robust hierarchy across exercise modalities. Therefore, ranking results should be interpreted with caution.ndomized trials with sufficient direct head-to-head comparisons. Fifth, domain-specific cognitive effects should be interpreted cautiously because of task impurity in neuropsychological assessment. Cognitive tasks often involve multiple processes rather than a single pure domain; for example, executive function tasks may also require processing speed, attention, visuomotor coordination, and working memory. Thus, the pooled effects should be interpreted as domain-related rather than domain-pure effects.

## 5 Conclusion

This study suggests that exercise interventions can improve executive function, memory, and processing speed in cognitively healthy older adults, may provide modest benefits for working memory, but show no clear evidence of improvement in verbal fluency. Training frequency may represent an important prescription component associated with improvements in executive function and processing speed, and NMA findings further indicate that AE may have a favorable trend for executive and memory outcomes. Given the remaining uncertainty in between-modality comparisons, further well-designed randomized trials with direct head-to-head comparisons and long-term follow-up are required to confirm these findings.

## Data Availability

The original contributions presented in the study are included in the article/supplementary material, further inquiries can be directed to the corresponding author.

## Glossary

RCTs: randomized controlled trials
SMDs: standardized mean differences
NMA: network meta-analysis
AE: aerobic exercise
MBE: mind–body exercise
AC: active control
PC: passive control
MCI: mild cognitive impairment
RT: resistance training
MCE: multicomponent exercise
BT: balance training
WOS: Web of Science
MeSH: Medical Subject Headings
RoB 2: Risk of Bias 2
CrIs: credible intervals
SEs: standard errors
MD: mean difference
CIs: confidence intervals
UME: unrelated mean effects
DIC: deviance information criterion
PSRF: potential scale reduction factor

## References

[1] July, W. H. O. J. R. T. Risk Reduction of Cognitive Decline and Dementia: WHO Guidelines. Geneva: World Health Organization (2019). p. 13–45. 31219687

[2] Organization, W. H. Global, regional, and national burden of Alzheimer's disease and other dementias, 1990-2016: a systematic analysis for the global burden of disease study 2016. Lancet Neurol. (2019) 18:88–106. doi: 10.1016/S1474-4422(18)30403-4 30497964 PMC6291454

[3] Organization, W. H. (2025). Ageing and health. Available online at: https://www.who.int/news-room/fact-sheets/detail/ageing-and-health (Accessed October 20, 2025).

[4] Sperling RA Aisen PS Beckett LA Bennett DA Craft S Fagan AM . Toward defining the preclinical stages of Alzheimer's disease: recommendations from the National Institute on Aging-Alzheimer's Association workgroups on diagnostic guidelines for Alzheimer's disease. Alzheimers Dement. (2011) 7:280–92. doi: 10.1016/j.jalz.2011.03.003 21514248 PMC3220946

[5] Cummings J Zhou Y Lee G Zhong K Fonseca J Cheng F. Alzheimer's disease drug development pipeline: 2023. Alzheimer's & Dementia: Translational Res Clin Interventions. (2023) 9:e12385. doi: 10.1002/trc2.12385, 37251912 PMC10210334

[6] Jack CR Jr Bennett DA Blennow K Carrillo MC Dunn B Haeberlein SB . NIA-AA research framework: toward a biological definition of Alzheimer's disease. Alzheimers Dement. (2018) 14:535–62. doi: 10.1016/j.jalz.2018.02.018, 29653606 PMC5958625

[7] Livingston G Huntley J Liu KY Costafreda SG Selbæk G Alladi S . Dementia prevention, intervention, and care: 2024 report of the lancet standing commission. Lancet. (2024) 404:572–628. doi: 10.1016/S0140-6736(24)01296-0, 39096926

[8] Aisen PS Vellas B Hampel H. Moving towards early clinical trials for amyloid-targeted therapy in Alzheimer's disease. Nat Rev Drug Discov. (2013) 12:324. doi: 10.1038/nrd3842-c1, 23493086

[9] Cummings J Lee G Zhong K Fonseca J Taghva K. Alzheimer's disease drug development pipeline: 2021. Alzheimers Dement (N Y). (2021) 7:e12179. doi: 10.1002/trc2.12179, 34095440 PMC8145448

[10] Erickson KI Hillman C Stillman CM Ballard RM Bloodgood B Conroy DE . Physical activity, cognition, and brain outcomes: a review of the 2018 physical activity guidelines. Med Sci Sports Exerc. (2019) 51:1242–51. doi: 10.1249/MSS.0000000000001936, 31095081 PMC6527141

[11] Iso-Markku P Aaltonen S Kujala UM Halme H-L Phipps D Knittle K . Physical activity and cognitive decline among older adults: a systematic review and Meta-analysis. JAMA Netw Open. (2024) 7:e2354285. doi: 10.1001/jamanetworkopen.2023.54285, 38300618 PMC10835510

[12] Iso-Markku P Kujala UM Knittle K Polet J Vuoksimaa E Waller K. Physical activity as a protective factor for dementia and Alzheimer's disease: systematic review, meta-analysis and quality assessment of cohort and case-control studies. Br J Sports Med. (2022) 56:701–9. doi: 10.1136/bjsports-2021-104981, 35301183 PMC9163715

[13] Turner DT Hu MX Generaal E Bos D Ikram MK Heshmatollah A . Physical exercise interventions targeting cognitive functioning and the cognitive domains in nondementia samples: a systematic review of Meta-analyses. J Geriatr Psychiatry Neurol. (2021) 34:91–101. doi: 10.1177/0891988720915523, 32295450 PMC7859677

[14] Colcombe S Kramer AF. Fitness effects on the cognitive function of older adults: a meta-analytic study. Psychol Sci. (2003) 14:125–30. doi: 10.1111/1467-9280.t01-1-01430, 12661673

[15] Erickson KI Voss MW Prakash RS . Exercise training increases size of hippocampus and improves memory. Proc Natl Acad Sci U S A, (2011) 108, 3017–22. doi: 10.1073/pnas.1015950108 21282661 PMC3041121

[16] Feter N Feter J Silva GS Schmidt MI Rombaldi AJ. Physical activity: a neglected therapy for dementia. Cad Saude Publica. (2024) 40:e00216123. doi: 10.1590/0102-311xen216123, 39442161 PMC11488822

[17] Gholami F Mesrabadi J Iranpour M Donyaei A. Exercise training alters resting brain-derived neurotrophic factor concentration in older adults: a systematic review with meta-analysis of randomized-controlled trials. Exp Gerontol. (2025) 199:112658. doi: 10.1016/j.exger.2024.112658, 39674562

[18] Hillman CH Erickson KI Kramer AF. Be smart, exercise your heart: exercise effects on brain and cognition. Nat Rev Neurosci. (2008) 9:58–65. doi: 10.1038/nrn2298, 18094706

[19] Hasher L Zacks RT. "Working memory, comprehension, and aging: a review and a new view". In: Bower GH, editor. Psychology of Learning and Motivation. New York, NY, USA: Academic Press (1988)

[20] Salthouse TA. The processing-speed theory of adult age differences in cognition. Psychol Rev. (1996) 103:403–28. 8759042 10.1037/0033-295x.103.3.403

[21] Wu J Huang C. A systematic review and meta-analysis of the effects of resistance exercise on cognitive function in older adults. Front Psychol. (2025) 16:1708244. doi: 10.3389/fpsyt.2025.1708244, 41503279 PMC12772445

[22] Xu L Gu H Cai X Zhang Y Hou X Yu J . The effects of exercise for cognitive function in older adults: a systematic review and meta-analysis of randomized controlled trials (2023). Int. J. Environ. Res. Public Health. 20:1088. doi: 10.3390/ijerph20021088 PMC9858649 36673844

[23] Au J Gibson BC Bunarjo K Buschkuehl M Jaeggi SM. Quantifying the difference between active and passive control groups in cognitive interventions using two Meta-analytical approaches. J Cogn Enhanc. (2020) 4:192–210. doi: 10.1007/s41465-020-00164-6, 34337311 PMC8320766

[24] Chen W Siew-Pin JL Wu Y Huang N Teo WP. Identifying exercise and cognitive intervention parameters to optimize executive function in older adults with mild cognitive impairment and dementia: a systematic review and meta-analyses of randomized controlled trials. Eur Rev Aging Phys Act. (2024) 21:22. doi: 10.1186/s11556-024-00357-4, 39215230 PMC11363393

[25] Han C Sun W Zhang D Xi X Zhang R Gong W. Effects of different aerobic exercises on the global cognitive function of the elderly with mild cognitive impairment: a meta-analysis. BMJ Open. (2023) 13:e067293. doi: 10.1136/bmjopen-2022-067293, 37399446 PMC10314475

[26] Lu H-H Zhou Y Chen C Gu Z-J. Meta-analysis of the effect of exercise intervention on cognitive function in elderly patients with type 2 diabetes mellitus. BMC Geriatr. (2024) 24:770. doi: 10.1186/s12877-024-05352-z, 39300333 PMC11411744

[27] Nikolakopoulou A Higgins JPT Papakonstantinou T Chaimani A Del Giovane C Egger M . CINeMA: an approach for assessing confidence in the results of a network meta-analysis. PLoS Med. (2020) 17:e1003082. doi: 10.1371/journal.pmed.1003082, 32243458 PMC7122720

[28] Papakonstantinou T Nikolakopoulou A Higgins JPT Egger M Salanti G. CINeMA: software for semiautomated assessment of the confidence in the results of network meta-analysis. Campbell Syst Rev. (2020) 16:e1080. doi: 10.1002/cl2.1080, 37131978 PMC8356302

[29] Albinet CT Abou-Dest A André N Audiffren M. Executive functions improvement following a 5-month aquaerobics program in older adults: role of cardiac vagal control in inhibition performance. Biol Psychol. (2016) 115:69–77. doi: 10.1016/j.biopsycho.2016.01.010, 26812613

[30] Albinet CT Boucard G Bouquet CA Audiffren M. Increased heart rate variability and executive performance after aerobic training in the elderly. Eur J Appl Physiol. (2010) 109:617–24. doi: 10.1007/s00421-010-1393-y, 20186426

[31] Antunes HKM De Mello MT DE Aquino Lemos V Santos-Galduroz RF Galdieri LC Bueno OFA . Aerobic physical exercise improved the cognitive function of elderly males but did not modify their blood homocysteine levels. Dementia Geriatric Cognitive Disorders Extra. (2015) 5:13–24. doi: 10.1159/000369160, 25759715 PMC4335628

[32] Fabre C Chamari K Mucci P Masse-Biron J Prefaut C. Improvement of cognitive function by mental and/or individualized aerobic training in healthy elderly subjects. Int J Sports Med. (2002) 23:415–21. doi: 10.1055/s-2002-33735, 12215960

[33] Fairbairn P Tsofliou F Johnson A Dyall SC. Effects of a high-DHA multi-nutrient supplement and exercise on mobility and cognition in older women (MOBILE): a randomised semi-blinded placebo-controlled study. Br J Nutr. (2020) 124:146–55. doi: 10.1017/S0007114520000719, 32100647

[34] Ferreira L Tanaka K Santosgalduróz RF Galduróz JCF. Respiratory training as strategy to prevent cognitive decline in aging: a randomized controlled trial. Clin Interv Aging. (2015) 10:593–603. doi: 10.2147/CIA.S79560, 25848235 PMC4374650

[35] Galle SA Deijen JB Milders MV De Greef MHG Scherder EJA Van Duijn CM . The effects of a moderate physical activity intervention on physical fitness and cognition in healthy elderly with low levels of physical activity: a randomized controlled trial. Alzheimer's Res Ther. (2023) 15:12. doi: 10.1186/s13195-022-01123-3, 36631905 PMC9832427

[36] Gujral S Cameron JL Conaty K Ziady S Sahu A Jakicic JM . Intermittent low-intensity and moderate-intensity exercise effects on cognition in community-dwelling older adults: a pilot study exploring biological mechanisms. Front Aging Neurosci. (2024) 16:1432909. doi: 10.3389/fnagi.2024.1432909, 39484365 PMC11524916

[37] Iuliano E Di Cagno A Aquino G Fiorilli G Mignogna P Calcagno G . Effects of different types of physical activity on the cognitive functions and attention in older people: a randomized controlled study. Exp Gerontol. (2015) 70:105–10. doi: 10.1016/j.exger.2015.07.008, 26183691

[38] Maki Y Ura C Yamaguchi T Murai T Isahai M Kaiho A . Effects of intervention using a community-based walking program for prevention of mental decline: a randomized controlled trial. J Am Geriatr Soc. (2012) 60:505–10. doi: 10.1111/j.1532-5415.2011.03838.x 22288578

[39] Santos T Rocha S Vasconcelos L Queiroz B Oliveira S Coutinho AP. The effect of physical exercise on the memory of elderly - an intervention study. Motriz Rev Educ Fis. (2019) 25:e10190020. doi: 10.1590/S1980-6574201900040020

[40] Satoh M Ogawa J Tokita T Nakaguchi N Nakao K Kida H . The effects of physical exercise with music on cognitive function of elderly people: mihama-Kiho project. PLoS One. (2014) 9:e95230. doi: 10.1371/journal.pone.0095230, 24769624 PMC4000225

[41] Silva AG Martins AI Andias R Nery E Silva T Ribeiro Ó . A web step-based digital solution's impact on physical, cognitive and psychosocial functioning of community-dwelling older adults: a mixed methods randomized and controlled trial. Internet Interv. (2024) 38:100766. doi: 10.1016/j.invent.2024.100766, 39280041 PMC11393595

[42] Tomoto T Verma A Kostroske K Tarumi T Patel NR Pasha EP . One-year aerobic exercise increases cerebral blood flow in cognitively normal older adults. J Cereb Blood Flow Metab. (2023) 43:404–18. doi: 10.1177/0271678X221133861, 36250505 PMC9941859

[43] Varela S Cancela JM Seijo-Martinez M Ayan C. Self-paced cycling improves cognition on institutionalized older adults without known cognitive impairment: a 15-month randomized controlled trial. J Aging Phys Act. (2018) 26:1–29. doi: 10.1123/japa.2017-0135 29431549

[44] Wang L Guo F Zhao CX Zhao MM Zhao CL Guo JW . The effect of aerobic dancing on physical fitness and cognitive function in older adults during the COVID-19 pandemic-a natural experiment. Sports Med Health Sci. (2023) 5:196–204. doi: 10.1016/j.smhs.2023.07.005, 37753419 PMC10518797

[45] Welford P Östh J Hoy S L Rossell S Pascoe M Diwan V . Effects of yoga and aerobic exercise on verbal fluency in physically inactive older adults: randomized controlled trial (FitForAge). Clin Interv Aging. (2023) 18:533–45. doi: 10.2147/CIA.S359185 37021083 PMC10069432

[46] Zhao CX Zhao CL Zhao MM Wang L Guo JW Zhang LH . Effect of exergame training on working memory and executive function in older adults. Sustainability. (2022) 14:10631. doi: 10.3390/su141710631

[47] Hong S-G Kim J-H Jun T-W. Effects of 12-week resistance exercise on electroencephalogram patterns and cognitive function in the elderly with mild cognitive impairment: a randomized controlled trial. Clin J Sport Med. (2018) 28:500–8. doi: 10.1097/JSM.0000000000000476, 28727639

[48] Jansen P Dahmen-Zimmer K Kudielka BM Schulz A. Effects of karate training versus mindfulness training on emotional well-being and cognitive performance in later life. Res Aging. (2017) 39:1118–44. doi: 10.1177/0164027516669987, 27688143

[49] Klusmann V Evers A Schwarzer R Schlattmann P Reischies FM Heuser I . Complex mental and physical activity in older women and cognitive performance: a 6-month randomized controlled trial. J Gerontol A Biol Sci Med Sci. (2010) 65:680–8. doi: 10.1093/gerona/glq053, 20418350

[50] Shimada H Lee S Akishita M Kozaki K Iijima K Nagai K . Effects of golf training on cognition in older adults: a randomised controlled trial. J Epidemiol Community Health. (2018) 72:944–50. doi: 10.1136/jech-2017-210052, 29936419

[51] Stroehlein JK Vieluf S Zimmer P Schenk A Oberste M Goelz C . Learning to play golf for elderly people with subjective memory complaints: feasibility of a single-blinded randomized pilot trial. BMC Neurol. (2021) 21:200. doi: 10.1186/s12883-021-02186-9, 34001020 PMC8127313

[52] Gothe NP Kramer AF Mcauley E. Hatha yoga practice improves attention and processing speed in older adults: results from an 8-week randomized control trial. J Altern Complement Med. (2017) 23:35–40. doi: 10.1089/acm.2016.0185, 27809558

[53] Hariprasad VR Koparde V Sivakumar PT Varambally S Thirthalli J Varghese M . Randomized clinical trial of yoga-based intervention in residents from elderly homes: effects on cognitive function. Indian J Psychiatry. (2013) 55:S357–63. doi: 10.4103/0019-5545.116308, 24049199 PMC3768212

[54] Hola V Pozanska H Jandova T Dytrtová JJ Weinerova J Steffl M . The effect of two somatic-based practices dance and martial arts on Irisin, BDNF levels and cognitive and physical fitness in older adults: a randomized control trial. Clin Interv Aging. (2024) 19:1829–42. doi: 10.2147/CIA.S482479, 39525874 PMC11550684

[55] Hong S Haina W Lina M Su H Wang H Meng L. The effects of Baduanjin exercise on the subjective memory complaint of older adults: a randomized controlled trial. Medicine. (2021) 100:1–8. 10.1097/MD.0000000000025442 PMC8322475 34397680

[56] Chen F-T Etnier JL Chan K-H Chiu P-K Hung T-M Chang Y-K. Effects of exercise training interventions on executive function in older adults: a systematic review and Meta-analysis. Sports Med. (2020) 50:1451–67. doi: 10.1007/s40279-020-01292-x, 32447717 PMC7376513

[57] Ye M Song T Xia H Hou Y Chen A. Effects of aerobic exercise on executive function of healthy middle-aged and older adults: a systematic review and meta-analysis. Int J Nurs Stud. (2024) 160:104912. doi: 10.1016/j.ijnurstu.2024.104912, 39326271

[58] Zhang M Jia J Yang Y Zhang L Wang X. Effects of exercise interventions on cognitive functions in healthy populations: a systematic review and meta-analysis. Ageing Res Rev. (2023) 92:102116. doi: 10.1016/j.arr.2023.102116, 37924980

[59] Yu F Vock DM Barclay TR. Executive function: responses to aerobic exercise in Alzheimer's disease. Geriatr Nurs. (2018) 39:219–24. doi: 10.1016/j.gerinurse.2017.09.005, 29031520 PMC5897173

[60] Han H Zhang J Zhang F Li F Wu Z. Optimal exercise interventions for enhancing cognitive function in older adults: a network meta-analysis. Front Aging Neurosci. (2025):2025:17. doi: 10.3389/fnagi.2025.1510773 PMC12289702 40717897

[61] Di Lorito C Bosco A Booth V Goldberg S Harwood RH. Adherence to exercise interventions in older people with mild cognitive impairment and dementia: a systematic review and meta-analysis. Prev Med Rep. (2020) 19:101139. doi: 10.1016/j.pmedr.2020.101139, 32793408 PMC7414005

[62] Bai Y Yuan Y Qiu B Yang Y Cheng C Wang J . Effects of different non-pharmacological interventions on executive function in healthy older people: a systematic review and network meta-analysis. Arch Gerontol Geriatr. (2026) 143:106141. doi: 10.1016/j.archger.2026.106141, 41576774

[63] Northey JM Cherbuin N Pumpa KL Smee DJ Rattray B. Exercise interventions for cognitive function in adults older than 50: a systematic review with meta-analysis. Br J Sports Med. (2018) 52:154–60. doi: 10.1136/bjsports-2016-096587, 28438770

[64] Nagamatsu LS Chan A Davis JC Beattie BL Graf P Voss MW . Physical activity improves verbal and spatial memory in older adults with probable mild cognitive impairment: a 6-month randomized controlled trial. J Aging Res. (2013) 2013:861893. doi: 10.1155/2013/861893 23509628 PMC3595715

[65] Chen R Zhao B Huang J Zhang M Wang Y Fu J . The effects of different exercise interventions on patients with subjective cognitive decline: a systematic review and network Meta-analysis. J Prev Alzheimers Dis. (2024) 11:620–31. doi: 10.14283/jpad.2024.65, 38706278 PMC11060994

[66] Vaportzis E Niechcial MA Gow AJ. A systematic literature review and meta-analysis of real-world interventions for cognitive ageing in healthy older adults. Ageing Res Rev. (2019) 50:110–30. doi: 10.1016/j.arr.2019.01.006, 30707947

[67] Wu Y Zang M Wang B Guo W. Does the combination of exercise and cognitive training improve working memory in older adults? A systematic review and meta-analysis. PeerJ. (2023) 11:e15108. doi: 10.7717/peerj.15108, 37065695 PMC10100799

[68] Wu C Yi Q Zheng X Cui S Chen B Lu L . Effects of mind-body exercises on cognitive function in older adults: a Meta-analysis. J Am Geriatr Soc. (2019) 67:749–58. doi: 10.1111/jgs.15714, 30565212

[69] Suk JW Kim K Kim JU. A Meta-analysis of studies of the effect of mind body exercise on various domains of cognitive function in older people with or without mild cognitive impairment. J Evid Based Integr Med. (2025) 30:2515690x251363709. doi: 10.1177/2515690X251363709, 40767037 PMC12329208

[70] Smith PJ Blumenthal JA Hoffman BM Cooper H Strauman TA Welsh-Bohmer K . Aerobic exercise and neurocognitive performance: a meta-analytic review of randomized controlled trials. Psychosom Med. (2010) 72:239–52. doi: 10.1097/PSY.0b013e3181d14633, 20223924 PMC2897704

[71] Boa Sorte Silva NC Barha CK Erickson KI Kramer AF Liu-Ambrose T. Physical exercise, cognition, and brain health in aging. Trends Neurosci. (2024) 47:402–17. doi: 10.1016/j.tins.2024.04.004 38811309

[72] Guadagni V Drogos LL Tyndall AV Davenport MH Anderson TJ Eskes GA . Aerobic exercise improves cognition and cerebrovascular regulation in older adults. Neurology. (2020) 94:e2245–57. doi: 10.1212/WNL.0000000000009478, 32404355 PMC7357295

[73] Dhahbi W Briki W Heissel A Schega L Dergaa I Guelmami N . Physical activity to counter age-related cognitive decline: benefits of aerobic, resistance, and combined training-a narrative review. Sports Med Open. (2025) 11:56. doi: 10.1186/s40798-025-00857-2, 40381170 PMC12085549

[74] Jandova T Buendía-Romero A Polanska H Hola V Rihova M Vetrovsky T . Long-term effect of exercise on Irisin blood levels-systematic review and Meta-analysis. Healthcare (Basel). (2021) 9:1438. doi: 10.3390/healthcare9111438 34828485 PMC8618299

[75] Jimenez-Roldán MJ Sañudo Corrales B Carrasco Páez L. Effects of high-intensity interval training on executive functions and IGF-1 levels in sedentary young women: a randomized controlled trial. Front Sports Act Living. (2025) 7:1597171. doi: 10.3389/fspor.2025.1597171, 40538943 PMC12177337

[76] Stillman CM Esteban-Cornejo I Brown B Bender CM Erickson KI. Effects of exercise on brain and cognition across age groups and health states. Trends Neurosci. (2020) 43:533–43. doi: 10.1016/j.tins.2020.04.010, 32409017 PMC9068803

[77] Kandola A Ashdown-Franks G Hendrikse J Sabiston CM Stubbs B. Physical activity and depression: towards understanding the antidepressant mechanisms of physical activity. Neurosci Biobehav Rev. (2019) 107:525–39. doi: 10.1016/j.neubiorev.2019.09.040, 31586447

[78] Singh B Olds T Curtis R Dumuid D Virgara R Watson A . Effectiveness of physical activity interventions for improving depression, anxiety and distress: an overview of systematic reviews. Br J Sports Med. (2023) 57:1203–9. doi: 10.1136/bjsports-2022-106195 36796860 PMC10579187

